# Prolactin-Secreting Leiomyoma Causing Hyperprolactinaemia Unresponsive to Dopamine Agonist Therapy and Resolution following Myomectomy

**DOI:** 10.1155/2021/5553187

**Published:** 2021-09-09

**Authors:** Lucinda Barry, Selvan Pather, Ash Gargya, Anthony Marren

**Affiliations:** ^1^Royal Prince Alfred Hospital, Sydney, Australia; ^2^Chris O'Brien Lifehouse, Sydney, Australia

## Abstract

Prolactin-secreting leiomyomas are rare, with only eight cases reported in the literature. This case describes a 37-year-old female with hyperprolactinaemia (1846 mIU/L; 85–500 mIU/L) refractory to cabergoline causing infertility and galactorrhea. MRI pituitary was normal. The patient had a known enlarging uterine leiomyoma on serial pelvic ultrasounds (15.2 cm × 9.1 cm × 12.1 cm). The serum prolactin returned to subnormal levels two days postmyomectomy and showed recovery to normal levels in the months following surgery. Immunostaining of the leiomyoma for prolactin was negative. Despite not staining for prolactin, quick resolution of the patient's hyperprolactinaemia after myomectomy supports the diagnosis of a prolactin-secreting fibroid. A prolactin-secreting leiomyoma should be considered in patients with hyperprolactinaemia and normal pituitary MRI which is refractory to dopamine receptor agonist therapy who also have evidence of a uterine fibroid. In patients wishing to seek fertility, myomectomy should be considered to allow for normal ovulation and possibility of future fertility.

## 1. Introduction

Leiomyomas (uterine fibroids) are common, noncancerous smooth muscle tumours which originate from uterine myometrial cells and are estimated to affect 40–80% of women [1]. Leiomyomas are often asymptomatic and incidentally diagnosed on pelvic ultrasound. However, they can cause abnormal uterine bleeding, pain, mass effect symptoms (urinary or bowel), reduced fertility, and obstetric complications [1]. The cause of leiomyomas is generally unknown; they are, however, known to have oestrogen and progesterone receptors, and their growth is thought to be promoted by these hormones [1]. Leiomyomas typically do not secrete hormones.

Prolactin is a hormone secreted from the anterior pituitary which is regulated by the hypothalamus [2]. It is primarily responsible for stimulating lactation in women postpartum [2].

Prolactin secretion inhibits secretion of gonadotropin-releasing hormone (GnRH) from the hypothalamus which in turn inhibits secretion of follicle-stimulating hormone (FSH) and luteinizing hormone (LH) from the pituitary. FSH and LH are essential in reproduction. In women, a decrease in LH can inhibit ovulation and secretion of oestrogen and progesterone from the ovaries, causing infertility [2].

Prolactin is under tonic inhibition by dopamine that is secreted by the hypothalamus. Pathological hyperprolactinaemia is most commonly caused by a prolactin-secreting pituitary adenoma [2]. Patients with hyperprolactinaemia are often managed with dopamine receptor agonists in order to decrease production of prolactin [2].

We present a case of a patient with leiomyoma who had hyperprolactinaemia unresponsive to medical therapy. The hyperprolactinaemia resolved postresection of her leiomyoma.

## 2. Case Report

A 37-year-old female underwent investigation for infertility. The patient and her partner were referred to a fertility specialist for review after 14 months of unprotected intercourse without conceiving. Routine fertility investigations of the patient's partner were normal. Her menstrual cycles were irregular (28–75 days). The patient was not taking any regular medications and had no known medical conditions.

**Figure 1:**
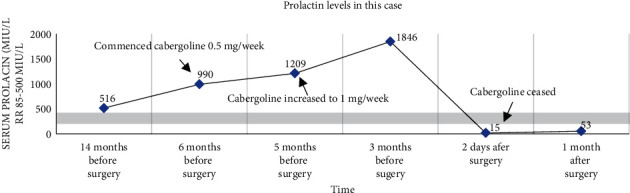
Prolactin levels before and after surgery in this case.

Initial routine investigations revealed a mildly elevated prolactin level of 516 mIU/L (85–500 mIU/L), which was not to be significant. TSH was normal. FSH, LH, oestradiol, and progesterone were normal (Table 1). Her antral follicle count was 17 and 19, so she was commenced on letrozole for ovulation induction. The patient did two cycles of letrozole ovulation induction which resulted in ovulation. She developed galactorrhea with a repeat prolactin 990 mIU/L (Figure 1). There was no evidence of interfering macroprolactin on post-PEG (polyethylene glycol) with a recovery of 97%. A post-PEG recovery of greater than 60% excludes the presence of a significant macroprolactin component. MRI pituitary was normal.

A pelvic ultrasound was performed as part of the routine investigations for infertility. This revealed a subserosal leiomyoma measuring 10.3 × 7.2 × 10.5 cm (446 mL). No significant endometrial cavity abnormality was detected.

The patient was commenced on cabergoline; despite increasing the dose of cabergoline to 1 mg/week, the serum prolactin continued to rise to 1856 mIU/L (Figure 1). There was no change to her menstrual cycle with cabergoline.

A repeat pelvic ultrasound was performed a few months later for the suspicion of ectopic prolactin secretion from the leiomyoma given the refractory hyperprolactinaemia. The ultrasound demonstrated significant enlargement of the uterine mass to 15.2 × 9.1 × 12.1 cm (876 mL) with cystic changes and increased vascularity. Sarcomatous change could not be excluded. A subsequent MRI pelvis showed there was no surrounding invasion, so a leiomyosarcoma was thought to be less likely; however, due to the marked increase in size, leiomyosarcoma could not be excluded.

**Figure 2:**
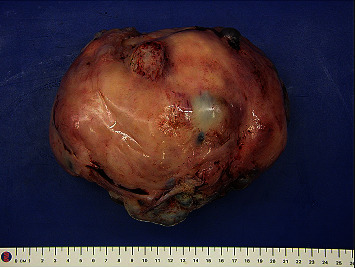
Macroscopic image of leiomyoma.

**Figure 3:**
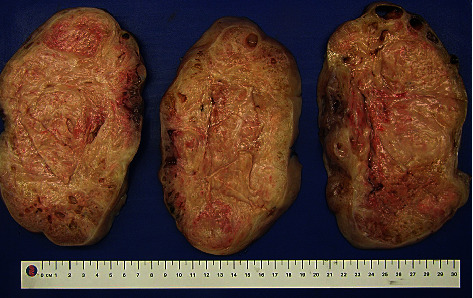
Macroscopic image of leiomyoma—cross-section.

The patient underwent a hysteroscopy, endometrial biopsy, laparoscopy, midline laparotomy, and myomectomy. Saline demonstrated normal ostia bilaterally, normal uterine cavity, and normal endocervical canal. The tubes and ovaries appeared normal bilaterally. There was no evidence of endometriosis. A single port laparoscopy at Palmer's point demonstrated a large pedunculated fundal fibroid. This was converted to a midline laparotomy which revealed a 20 cm vascular pedunculated fibroid, and a myomectomy was performed (Figures 2 and 3).

Histopathology confirmed a benign uterine leiomyoma. There were prominent ischaemic degenerated hydropic and hyaline changes seen as well as degenerative and haemorrhagic areas. Evidence of serosal and subserosal endometriosis with adhesions was also seen on histopathology. Immunostaining of the leiomyoma for prolactin was negative.

Her serum prolactin level two days postoperatively had significantly decreased to subnormal levels (Figure 1).

The patient recovered well postoperatively and is planning to commence IVF in the coming months. Her prolactin levels showed recovery towards normal reference range one-month postsurgery.

## 3. Discussion

This case described a 37-year-old female with hyperprolactinaemia found when investigated for a cause of galactorrhea and infertility. She had a rising prolactin level despite an increasing dose of cabergoline and a normal pituitary MRI. She was found to have an enlarging uterine leiomyoma measuring 15.2 × 9.1 × 12.1 cm. The hyperprolactinaemia rapidly decreased after myomectomy.

There are 8 cases in literature reporting resolution of hyperprolactinaemia after removal of a uterine mass [3–10]. Six of the eight cases revealed leiomyomas on histopathology [3, 5–9]. Of the two other cases, histopathology revealed a low-grade mesenchymal tumour [4] and a uterine tumour resembling ovarian sex cord tumour [10]. Of the six leiomyomas reported, all women were premenopausal (25–47 years). All had no evidence of pituitary adenoma on MRI imaging. The time to diagnosis was 6 months up to 10 years from initial presentation, and the most common presenting complaint was an irregular menstrual cycle. All patients were treated with dopamine agonist therapy, either cabergoline or bromocriptine. The prolactin level continued to rise despite this in all cases (Table 2).

The serum prolactin levels in all 6 patients returned to normal after removal of the leiomyomas, either through hysterectomy or myomectomy, which support the diagnosis of ectopic secretion of prolactin. However, of the 6 leiomyomas reported, only one case reported positive immunostaining for prolactin [3]. Four cases, including this case, did not exhibit immunostaining for prolactin [7–9], and two were not reported [5, 6].

In addition to the anterior pituitary, prolactin has been found to be synthesised and secreted by the uterine myometrium, decidua cells, and leiomyomas [11, 12]. There is evidence that prolactin from decidualised endometrial stroma has an important role in maintaining healthy pregnancies, while myometrial prolactin likely plays a role in maintaining regular menstrual cycles [13]. Two studies found that prolactin acts as an autocrine or paracrine growth factor for leiomyomas and myometrial cells, promoting their proliferation [14, 15]. However, each leiomyoma has been found to have different sensitivities to prolactin [16]. Another study found that prolactin secretion from leiomyoma is greater than myometrial prolactin secretion in the same patient [17]. In addition, prolactin secretion from a leiomyoma increases with time, whereas myometrial prolactin did not. The study also found that as prolactin secretion from a leiomyoma increased, the prolactin receptors on the leiomyoma also increased [17], which likely contributed to growth.

Dopamine receptors have also been found on leiomyomas, with overexpression of dopamine receptors correlating to proliferation of leiomyomas [18]. A study of patients with leiomyomas but no evidence of serum hyperprolactinaemia showed statistically significant shrinkage of the tumour with 0.5 mg of cabergoline once weekly for 6 weeks [19], confirming the presence of dopamine receptors. Another study found significant improvement in symptoms related to leiomyoma like per vaginal bleeding and pain, as well as a decrease in the size of the leiomyoma with cabergoline 0.5 mg per week for 3 months. All patients's serum prolactin was tested prior to the study and found to be normal [16]. The leiomyoma in this case did not respond to either cabergoline. The leiomyomas in the other case reports also did not respond to dopamine agonist therapy. Letrozole (aromatase inhibitor) has also been reported to shrink the size of leiomyomas [18]. Letrozole in this case was only used for five days for ovulation induction, so it was not expected to affect the leiomyoma in this case.

Some leiomyomas appear to be more sensitive to prolactin as a growth factor causing unregulated growth and clinically significant hyperprolactinaemia. Complete resistance or paradoxical rise on dopamine agonists may act as a red flag for ectopic prolactin secretion. As discussed, leiomyomas with increased prolactin secretion also had increased prolactin receptors. Dopamine receptors have been found on leiomyomas, and shrinkage of some has been seen with dopamine agonist therapy in patients without hyperprolactinaemia. The leiomyoma in this case was sensitive to prolactin and hence continued to proliferate until hyperprolactinaemia was evident. As discussed, high levels of prolactin caused an increase in prolactin receptors on leiomyomas. It is plausible that while this leiomyoma may have had dopamine receptors, the unregulated proliferation of prolactin and significantly increased expression of prolactin receptors meant that it did not respond to dopamine agonist therapy. Further research is required to further determine the underlying cause of this phenomenon and diagnostic indicators.

With regards to pituitary prolactinomas, serum prolactin is generally directly proportionate to the size of the tumour, with a tumour >4 cm correlating to a serum prolactin level of ≥5276 mIU/L [20]. Each of the six cases including this case reported large leiomyomas >8 cm [3, 9], but all with moderately raised prolactin levels (≤5900 mIU/L). This suggests the leiomyomas are likely poorly differentiated with only small focal areas secreting prolactin. This is confirmed by the case which was positive when immunostaining for prolactin as it reported “focal but intense” positive areas of prolactin-secreting cells [3]. It is possible that the cases where immunostaining was negative also had small focal areas of prolactin-secreting cells which were not seen in the samples. This may have been complicated by the areas of haemorrhage and ischaemic changes which were seen on histopathology in this case.

The serum prolactin levels of each case were not directly proportionate to the size of the leiomyoma and could not be correlated to immunostaining positivity (Table 2).

This case is significant, in that it supports the current few reports of ectopic secreting prolactin from a leiomyoma. Despite not staining for prolactin, the patient's prolactin level decreased to subnormal levels within two days postoperatively. This was due to suppression of anterior pituitary lactotroph cells from ectopic hyperprolactinaemia. The prolactin then normalised. Quick resolution of the patient's hyperprolactinaemia after myomectomy supports the diagnosis of a prolactin-secreting fibroid.

A prolactin-secreting leiomyoma should be considered in patients with hyperprolactinaemia and normal pituitary MRI which is refractory to dopamine receptor agonist therapy who also have evidence of a uterine fibroid. In patients wishing to seek fertility, myomectomy should be considered to allow for normal ovulation and possibility of future fertility.

## Figures and Tables

**Table 1:** Hormone levels of patient before cabergoline in this case.

	Before cabergoline
FSH (basal RR 1.5–10 IU/L)	5.6 IU/L
LH (basal RR 2.0–12 IU/L)	5.5 IU/L
Oestradiol (follicular RR < 320 pmol/L)	108 pmol/L
Progesterone (follicular RR 0.3–4.0 nmol/L)	0.6 nmol/L

**Table 2:** Published case reports of ectopic secretion of prolactin from uterine leiomyomas.

Article reference	Age of patient	First clinical manifestations	Peak prolactin reported (mIU/L)	Dopamine agonist therapy	Dose cabergoline	Prolactin response	Histopathology	Size of leiomyoma	Time to diagnosis	Immunostaining for prolactin
[3]	47	Galactorrhoea	4765	Cabergoline	2 mg/week	Refractory	Leiomyoma	13.9 × 10.4 × 11.8 cm	10 months	Positive
[5]	44	Amenorrhoea	4638	Bromocriptine	30 mg/day	Refractory	Leiomyoma	8 × 7 cm	10 years	Not reported
[6]	36	Irregular menstrual cycle, galactorrhea, and headaches	2127	Bromocriptine	15 mg/day	Refractory	Leiomyoma	5.5 cm	6 months	Not reported
[7]	25	Irregular menstrual cycle and galactorrhoea	3191	Cabergoline bromocriptine	Dose not specified	Refractory	Leiomyoma	6 × 7.2 × 8 cm	3 years	Negative
[8]	45	Not reported	Not reported	Bromocriptine	Not reported	Refractory	Leiomyoma	9 cm	6 months	Negative
[9]	41	Amenorrhea and galactorrhea	5900	Dopamine agonist—not specified	Not reported	Refractory	Leiomyoma	Not reported	6 years	Negative
This case	37	Irregular menstrual cycle and galactorrhea	1846	Cabergoline	1 mg/week	Refractory	Leiomyoma	15.2 cm × 9.1 cm × 12.1 cm	14 months	Negative

## Data Availability

Data not applicable to this article as no datasets were generated or analysed during the current study.
